# Advancements in Artificial Intelligence for Kidney Transplantology: A Comprehensive Review of Current Applications and Predictive Models

**DOI:** 10.3390/jcm14030975

**Published:** 2025-02-03

**Authors:** Jakub Mizera, Maciej Pondel, Marta Kepinska, Patryk Jerzak, Mirosław Banasik

**Affiliations:** 1 Department of Nephrology and Transplantation Medicine, Institute of Internal Diseases, Wroclaw Medical University, 50-551 Wroclaw, Poland; patryk.jerzak@umw.edu.pl (P.J.); m.banasik@interia.pl (M.B.); 2 Department of Business Intelligence in Management, Wroclaw University of Economics and Business, 118-120 Komandorska St., 53-345 Wroclaw, Poland; maciej.pondel@ue.wroc.pl; 3 Department of Pharmaceutical Biochemistry, Faculty of Pharmacy, Wroclaw Medical University, Borowska 211a, 50-556 Wroclaw, Poland; marta.kepinska@umw.edu.pl

**Keywords:** artificial intelligence, deep learning, machine learning, nephrology, transplantology

## Abstract

**Background:** Artificial intelligence is rapidly advancing within the domains of medicine and transplantology. In this comprehensive review, we provide an in-depth exploration of current AI methodologies, with a particular emphasis on machine learning and deep learning techniques, and their diverse subtypes. These technologies are revolutionizing how data are processed, analyzed, and applied in clinical decision making. **Methods:** A meticulous literature review was conducted with a focus on the application of artificial intelligence in kidney transplantation. Four research questions were formulated to establish the aim of the review. **Results:** We thoroughly examined the general applications of AI in the medical field, such as feature selection, dimensionality reduction, and clustering, which serve as foundational tools for complex data analysis. This includes the development of predictive models for transplant rejection, the optimization of personalized immunosuppressive therapies, the algorithmic matching of donors and recipients based on multidimensional criteria, and the sophisticated analysis of histopathological images to improve the diagnostic accuracy. Moreover, we present a detailed comparison of existing AI-based algorithms designed to predict kidney graft survival in transplant recipients. In this context, we focus on the variables incorporated into these predictive models, providing a critical analysis of their relative importance and contribution to model performance. **Conclusions:** This review highlights the significant advancements made possible through AI and underscores its potential to enhance both clinical outcomes and the precision of medical interventions in the field of transplantology.

## 1. Introduction

Advanced chronic kidney disease (CKD) is one of the primary indications for renal replacement therapy. CKD, ranging from mild stages to end-stage renal disease, impacts around 10% of the global population, with many patients unaware of the progressive decline in their kidney function [1]. Kidney transplantation, considered the gold-standard treatment for those requiring renal replacement therapy, has been shown to result in substantially improved quality of life and higher survival rates compared to hemodialysis or other modalities. Although this method has numerous advantages, it is still necessary to establish immunological tolerance to preserve the transplanted organ [2].

Despite progress in immunosuppressive therapies, graft failure continues to be a major complication [3]. Due to the significant adverse outcomes associated with transplantation failure, it is crucial to investigate, identify, and mitigate the potential risk factors. Numerous studies have examined various prognostic factors influencing renal graft success, including donor and recipient age, donor type (deceased or living), body mass index, anemia presence, panel of reactive antibodies (PRA) level, immunosuppressive regimen, or histopathological features [4,5,6,7,8,9]. However, the findings across these studies have been inconsistent [10]. The development of novel technologies is essential to extend graft survival and to enhance the quality of life of transplant recipients. In the era of informatization, the integration of artificial intelligence (AI) represents substantial potential for advancing diagnostic and therapeutic approaches in kidney transplantation. AI algorithms can efficiently process and analyze large datasets obtained from patients who underwent the transplantation [11]. These algorithms excel in detecting subtle patterns and trends that may be missed by traditional methods, providing a more detailed understanding of the kidney graft rejection process. Furthermore, AI can be particularly valuable in integrating data from diverse domains of medical research, including clinical, molecular, and pathological datasets, thereby facilitating more comprehensive and holistic approaches to disease analysis and management [11].

AI methods introduced in kidney transplantation, such as machine learning (ML) or deep learning (DL), have facilitated the development of advanced tools. These include the AI-driven analysis of whole-slide images, which simplifies pathological evaluations, and predictive models that assess transplantation outcomes, enabling more personalized donor–recipient matching [12]. Nevertheless, as AI remains a still-developing field in medicine, there is a lack of standardization in these methods. The interdisciplinary application of AI in medicine, which merges the fields of information technology and clinical management, may present challenges for some physicians in terms of the effective utilization and comprehension. A survey conducted by Salvagno et al. revealed that nearly 25% of physicians expressed concerns regarding potential technical difficulties in the implementation of AI [13]. Consequently, we believe that review articles are essential to simplify and clarify the complexities surrounding AI in kidney transplantation, ensuring that the knowledge becomes accessible to both clinicians and researchers.

In this research, our objective is to synthesize the current body of knowledge regarding the implementation of AI technologies in renal transplantation. Additionally, we aimed to organize and summarize the findings of recent available studies to provide a comprehensive synthesis of the existing evidence on the application of AI in kidney transplantology.

## 2. Materials and Methods

**Figure 1:**
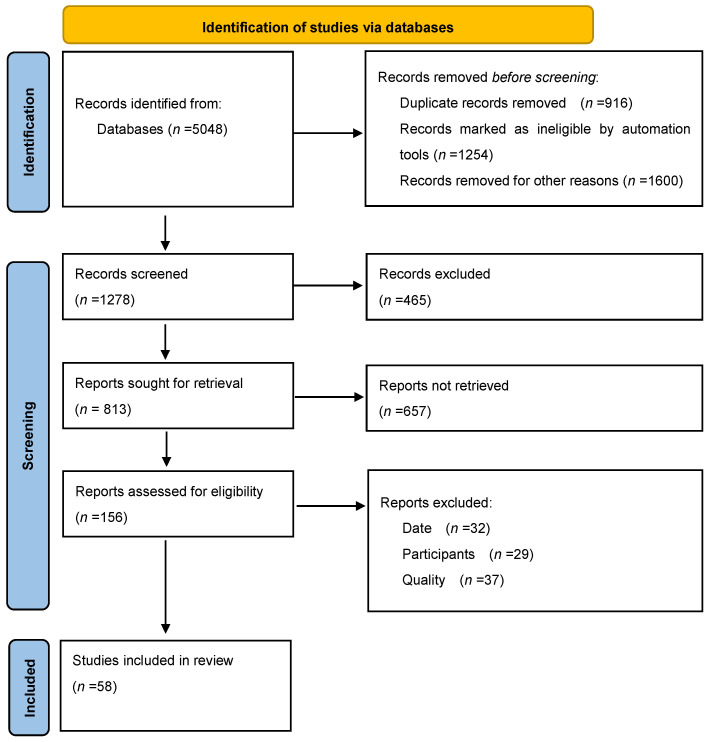
PRISMA flow diagram demonstrating the identification, selection, and inclusion of studies from databases.

In this literature review, the PRISMA (Preferred Reporting Items for Systematic Reviews and Meta-Analyses) guidelines were introduced to ensure high-quality reporting and to improve the methodological transparency. While the review does not meet all of the criteria for a systematic review, the PRISMA guidelines were only followed to structure the literature search, selection of studies, and assessment of the quality of the included sources (Figure 1) [14]. The details of the applied methodology are outlined as follows:

### 2.1. Literature Review—Methodology

The following research questions were formulated to guide the review, focusing on the application of machine learning in kidney transplantation, the specific areas of its application, and the key variables used in predictive models within this domain.

- RQ1: What machine learning (ML) algorithms and techniques have been applied in the context of kidney transplantation as reported in the literature?
- RQ2: What specific application areas of machine learning in kidney transplantation are identified in the literature?
- RQ3: Which method should be selected to achieve the most accurate results for the intended analysis?
- RQ4: What variables (features) are commonly used in predictive models related to kidney transplantation in the existing body of research?

The research questions were formulated using the PICO tool [15]. The PICO framework helps to develop a focused research question for a review by defining the following four key components: Population and Problem (P), Intervention of Interest (I), Comparison (C), and Outcome (O). In our review, the components were defined as follows:

P—Patients who are subjected to kidney transplantation;

I—Artificial intelligence utilization in pre- and post-transplantation interventions;

C—Traditional statistical models or human clinical judgment without AI support;

O—Improved prediction of transplant success, patient survival, and graft functionality; enhanced clinical decision making.

The following steps outline the process for conducting the literature review, aimed at addressing the three formulated research questions (RQ1, RQ2, RQ3, and RQ4):

(A) Defining the Sources for Literature Analysis

Identification of relevant databases, journals, and repositories that will serve as the primary sources of academic literature.

(B) Establishing the Search Strategy and Scope

Formulating a comprehensive search strategy using a combination of keywords, synonyms, and Boolean operators based on the research questions. Defining the scope of the search in terms of

- The time period;
- The type of publications (peer-reviewed journals and conference papers);
- The language.

(C) Selection Criteria for Inclusion

Developing clear inclusion and exclusion criteria for selecting scientific papers. Developer criteria included the following:

- Relevance to the research questions (RQ1, RQ2, and RQ3);
- Methodological rigor and quality of the studies;
- Theoretical or empirical focus;
- Availability of full-text versions for detailed analysis. Papers that meet these criteria will be retained for further analysis.

(D) Screening and Extraction of Relevant Papers

Conduct a two-stage screening process:

- Initial screening: Title and abstract review to eliminate papers that clearly do not meet the selection criteria.
- Detailed screening: Full-text analysis to ensure alignment with the research questions. Relevant papers will be extracted and compiled into a dataset for further analysis.

(E) Addressing Research Questions (RQ1, RQ2, and RQ3)

Extract relevant data from the selected studies to address each of the research questions.

(F) Synthesis of Results and Conclusion Formulation

Analyze the findings from the extracted studies to identify patterns, trends, and gaps. Synthesize these results in relation to each research question (RQ1, RQ2, and RQ3)

### 2.2. Literature Review—Findings

(A) Defining the Sources for Literature Analysis

A comprehensive literature search was conducted across multiple databases, including PubMed, Google Scholar, MEDLINE, and Scopus. These databases were selected due to their extensive coverage of medical and machine learning research relevant to kidney transplantation.

(B) Establishing the Search Strategy and Scope

The search strategy employed various combinations of keywords such as “machine learning”, “kidney transplantation”, “deep learning”, “artificial intelligence”, and “transplant survival”.

(C) Selection Criteria for Inclusion

Only peer-reviewed studies that addressed the application of machine learning in kidney transplantation were considered. The inclusion criteria included the relevance to the research questions, the publication date, and the availability of full-text articles. The methodological rigor and quality of the studies included in this analysis were assessed to ensure the reliability and validity of the findings. The quality assessment was performed in accordance with the criteria described by Qiao [16] and the risk of bias by the PROBAST tool [17]. Each study was evaluated based on predefined criteria, such as the study design, sample size, randomization, blinding, and data analysis methods. Studies with insufficient methodological transparency were excluded to maintain the integrity of the analysis. Studies not meeting these criteria, and articles with abstracts written in languages other than English and Polish, were excluded. The review included studies published up to December 2024.

(D) Screening and Extraction of Relevant Papers

The selection process began with a title and abstract screening to eliminate irrelevant studies. Following this, the remaining articles were subjected to full-text analysis to ensure alignment with the research focus. The most relevant studies were extracted for detailed review and data synthesis.

(E) Addressing Research Questions (RQ1, RQ2, and RQ3)

Data extracted from the selected studies were organized to address the following three primary research questions: the machine learning algorithms used (RQ1), the specific application areas in kidney transplantation (RQ2), and the variables identified in predictive models (RQ3).

(F) Synthesis of Results and Conclusion Formulation

The results from the extracted studies were analyzed and synthesized to identify key trends, patterns, and gaps in the literature.

## 3. Selection of AI Techniques and Machine Learning Algorithms

Examples of machine learning algorithms related to RQ1 demonstrated in our study were selected on the basis of quality assessment by criteria described by Qiao [16] and the risk of bias based on the PROBAST tool [17]. Models were assessed on the criteria which included the judgement assessment in non-machine learning limits, feature selection engineering, the platforms used, hyperparameters, validity of the methods, stability of the results, external validation, the predictors explained, and the suggested clinical use. Included studies were characterized by a high quality and low risk of bias [17].

Given the range of possibilities provided by AI, the RQ3 arises as follows: which method should be selected to achieve the most accurate results for the intended analysis?

At first, with hundreds of variables available, there are the methods which enable the selection of the most relevant features (e.g., Random Survival Forest (RSF) and Elastic Net, Boruta), which can be further used in the algorithms [18]. RSF is an ensemble-based machine learning method for survival analysis, handling time-to-event data and censoring. It extends the random forest algorithm to manage censored observations by constructing multiple decision trees and aggregating their predictions. RSF uses survival-specific splitting criteria, like the log-rank test, and is widely used due to its robustness and ability to handle high-dimensional data [19]. Elastic Net is a regularization technique in linear regression that combines Lasso (L1) and Ridge (L2) penalties. It balances feature selection and coefficient shrinkage, making it useful for datasets with highly correlated predictors or more variables than observations [20]. Boruta is a feature selection algorithm based on the random forest classifier. It identifies relevant features by comparing their importance to random “shadow” features. Boruta excels in high-dimensional datasets, distinguishing important variables even in complex, non-linear relationships [21].

After the variables are selected, the methods are used to perform clustering. It is a fundamental unsupervised machine learning technique aimed at identifying and grouping data points with similar characteristics based on their feature sets. The primary objective is to uncover inherent structures or patterns within a dataset without the use of predefined labels or classifications. Unlike supervised learning, where the model is trained using input–output pairs, clustering is exploratory in nature and seeks to discover underlying patterns [18].

The key components of clustering include the following [22]:

(A) Similarity Metrics: Clustering algorithms typically utilize mathematical measures of similarity or dissimilarity (e.g., Euclidean distance and cosine similarity) to quantify the relationship between data points. Points within a cluster are expected to exhibit higher similarity to one another compared to those in different clusters.
(B) Prominent Algorithms Selection: This allows for final feature combination selection, which maximizes the intercluster variability (e.g., Self-Organizing Map (SOM), Consensus Clustering, and Agglomerative Clustering). SOMs, also known as Kohonen maps, help to visualize and analyze high-dimensional data by creating a low-dimensional representation that preserves the data’s structure. Consensus Clustering improves the clustering accuracy by combining the results from multiple algorithms to produce a single, reliable solution. Agglomerative Clustering, a type of hierarchical clustering, merges the clusters based on their similarity, forming a hierarchy that can be visualized as a dendrogram.

**Figure 2:**
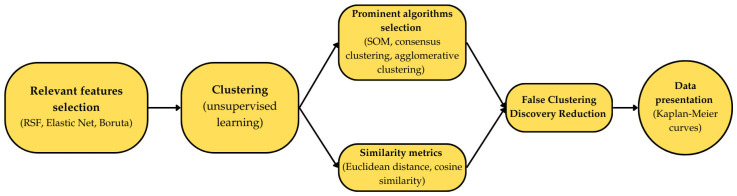
Graphical example of machine learning methods—from features selection to data presentation. (RSF—random survival forest; SOM—self-organizing map).

When the clustering is finished, the mechanism of False Clustering Discovery Reduction can be performed to achieve the smallest number of statistically differentiable populations. This allows us to verify that reducing the number of clusters does not result in a substantial loss of heterogeneity in the generated groupings. Further, final assessment can vary on the purpose of the analysis, and, for instance, when the survival of patients is to be assessed, the analysis can be performed with Kaplan–Meier curves [18] (Figure 2).

The aforementioned methods are only an example of a variety of techniques in ML. Other tools include support vector machines (SVMs), K-Nearest Neighbors (K-NNs), and Neural Networks (NNets) [23]. The SVM is a powerful supervised learning model used for classification and regression tasks, which works by finding a hyperplane that maximizes the margin between different classes in a dataset. It is effective in high-dimensional spaces and is particularly useful for binary classification problems [24]. Neural networks, inspired by the human brain, consist of interconnected layers of artificial neurons that learn to model complex, non-linear relationships in data. They are the foundation of deep learning and excel in tasks such as image recognition, natural language processing, and time series prediction. The K-NN, on the other hand, is a simple, instance-based algorithm used for both classification and regression. It classifies a data point based on the majority class of its nearest neighbors, making it computationally straightforward but sensitive to the choice of distance metrics and the number of neighbors. While the K-NN is easy to implement, it tends to be less scalable than the SVM or neural networks, especially with large datasets. Each of these methods has distinct advantages depending on the nature of the problem and dataset. Among these three methods, neural networks warrant particular attention in the context of kidney transplantation due to their capability to analyze medical images. In the future, they could serve as an assistive tool in renal pathology, enhancing the speed and accuracy of histopathological assessments [25,26].

## 4. AI Techniques in Kidney Transplantation—A Range of Applications

To address RQ3, we analyzed a diverse range of tools utilizing various AI techniques that have been developed for application in kidney transplantation. These tools are primarily based on machine learning and deep learning methodologies.

### 4.1. Machine Learning

Machine learning, a branch of artificial intelligence, involves the development of algorithms that autonomously recognize patterns within large datasets and learn from them with minimal direct human intervention. This data-driven approach holds significant promise for advancing renal transplantation by enhancing patient outcomes and optimizing the transplant process. In this context, ML models can predict critical post-transplant outcomes by analyzing intricate interactions between recipient health parameters, donor attributes, and immunological factors [27].

Numerous studies on the application of ML in renal transplantation have primarily focused on the following three key areas: predicting graft survival in both adult and pediatric populations, optimizing immunosuppressive drug dosing (e.g., tacrolimus), and improving the efficiency of donor–recipient matching [27,28,29,30].

#### 4.1.1. Predicting the Graft Survival

One of the key areas where ML excels is in predicting long-term graft survival. Traditional risk assessment tools, such as the Kidney Donor Risk Index (KDRI) or Estimated Post-Transplant Survival (EPTS) score, while widely employed, are constrained by their dependence on a limited set of clinical and demographic factors. These models do not account for the complexity of interactions between multiple variables. In contrast, ML models leverage extensive and diverse datasets, incorporating not only conventional clinical parameters but also genetic information, biomarkers, and even social determinants of health. This enables a more comprehensive and nuanced assessment of both donor and recipient profiles. By analyzing these multifaceted datasets, ML models provide a holistic evaluation, improving the accuracy of graft survival predictions and informing clinical decision making in transplantation [28,31].

ML models in renal transplantation exhibit considerable heterogeneity, influenced by various factors such as the feature selection method (FSE), the limitations of non-ML techniques, and the platforms used for model development. Additionally, the index tests employed to evaluate model performance differ significantly across studies. The primary distinction among these models lies in the clinical datasets used, including the sample size and the types of variables incorporated into the model construction. Most studies utilize preoperative donor and recipient data, while relatively few incorporate intraoperative and postoperative variables into their predictive frameworks. Moreover, many studies aim to predict graft survival at specific intervals (1, 3, or 5 years post-transplantation), with substantial variation in the models’ predictive accuracy across these time points, as evidenced by the wide prediction intervals reported [17,27,32].

Despite the observed variability in performance across the ML models, their overall and individual predictive capabilities are consistently reported to surpass those of established gold-standard tools like the KDRI. The KDRI demonstrates a reported C-index of 0.63, while most ML models exhibit better performance in predicting graft survival [17,33,34].

Evidence regarding the minimum sample size required to develop robust ML-based predictive models is limited. However, ML models built using both small single-center data and large databases have shown comparable predictive abilities, though larger datasets generally improve the performance. The model performance depends on factors such as the volume of clinical data, the complexity of the model, and the ML method used, with more events per variable linked to better stability and accuracy. The inclusion of clinically relevant variables is crucial for improving the predictive accuracy, making their identification a key step in model development [17]. Models which are currently developed mostly rely on variables such as the age and gender of the donor/recipient, HLA mismatch, cold/warm ischemia time, pre-transplant dialysis duration, comorbidities, serum creatinine levels, and the immunosuppressive therapy regimen [35,36].

To confirm that AI/ML models are reliable for predicting graft survival in kidney transplantation, strong external validation is necessary. Although these models perform well on the data they are trained on, many published models either lack external validation or have been poorly validated, which limits their broader use in practice [37,38].

#### 4.1.2. Immunosuppressive Agent Dosage Estimation

ML holds significant potential in the management of immunosuppressive therapy, a critical component in the success of kidney transplantation. Immunosuppressive agents are essential for preventing organ rejection; however, they pose considerable risks, such as infection and drug toxicity. By leveraging ML models, it is possible to optimize dosing regimens through the prediction of individual patient responses to specific immunosuppressive therapies. These predictions can be informed by genetic markers and other relevant clinical data, enabling a more personalized therapeutic approach. This method has the potential to reduce the adverse effects of immunosuppressive drugs while simultaneously lowering the risk of organ rejection [39]. In particular, very common immunosuppressive medications, which include calcineurin inhibitors such as tacrolimus and cyclosporin A, have narrow therapeutic windows, making precise dosing essential [40].

ML models support tacrolimus dose calibration by analyzing genetic polymorphisms, which include, for instance, CYP3A5—a key determinant of the drug’s metabolism. Additionally, these algorithms integrate patient-specific factors such as gender, age, and BMI, further refining predictions to account for individual variability [40]. Clinical studies have demonstrated that AI-powered tools were able to achieve the faster and more reliable attainment of target tacrolimus concentrations compared to traditional physician-guided dosing [41].

Similarly, cyclosporine A dosage optimization benefits from AI’s ability to account for complex pharmacokinetic factors, including liver enzyme activity, patient age, and drug interactions. Neural networks and adaptive systems have been used to predict the blood levels of cyclosporine with remarkable accuracy, guiding clinicians to safe and more effective dosing decisions [42].

#### 4.1.3. Donor–Recipient Pairing

Another area where ML can be effectively used is enhancing the efficiency of donor organ allocation by leveraging data from multiple transplant centers. These models analyze a range of factors, including donor organ quality, transport duration, and recipient urgency, to support more rapid and informed decisions regarding organ allocation. In the United States, ML algorithms have been incorporated into organ allocation systems to optimize the matching of recipients with donor kidneys that exhibit a high probability of successful outcomes. This application of ML is particularly beneficial in deceased donor transplants, where timely decision making is crucial [27]. For instance, neural network architectures such as DeepSurv are adept at modeling time-to-event data and predicting survival probabilities. These models demonstrate superior performance compared to traditional statistical approaches, such as Cox proportional hazards regression, in terms of both calibration and discriminative power. The utilization of such advanced neural networks enhances the precision of donor–recipient matching and informs clinicians more effectively about which organ offers the highest likelihood of long-term survival for individual recipients [27].

Despite the evident advantages of machine learning in the various aspects of renal transplantation, several challenges and limitations persist. One major issue is the necessity for extensive, high-quality datasets to develop accurate models, which can be difficult to obtain due to privacy concerns and inconsistent data collection practices across transplant centers. Additionally, there is a risk of algorithmic bias, where models may unintentionally perpetuate existing healthcare disparities by relying on historical data that reflects unequal access to care. To mitigate these challenges, researchers stress the importance of meticulously curating training datasets and ensuring transparency in the decision-making processes of machine learning models [31].

#### 4.1.4. Virtual Biopsy

The study by Yoo et al. introduced a novel machine learning-based virtual biopsy system aimed at predicting histological lesions in kidney transplant recipients by utilizing routinely available donor characteristics. A comprehensive analysis was performed on 14,032 protocol biopsies collected from 17 international centers, with a focus on the following four key types of renal injury: arteriosclerosis, arteriolar hyalinosis, interstitial fibrosis, and tubular atrophy. By employing six advanced machine learning algorithms, the model demonstrated robust predictive performance, as reflected by the high accuracy in both internal and external validation cohorts. These results highlight the potential of the virtual biopsy system to reduce interobserver variability in histopathological evaluations, refine organ allocation processes, and enhance patient monitoring and therapeutic decision making in kidney transplantation [43].

### 4.2. Deep Learning

Deep learning is a specialized branch of ML that uses artificial neural networks with multiple layers to model and process complex patterns in large datasets. Unlike traditional ML methods, which often require manual feature extraction and selection, DL models automatically learn hierarchical representations of data. They progress from low-level features to more abstract ones through their layers, making them particularly effective for handling unstructured data like images, audio, and text. DL is distinguished by its reliance on vast amounts of data and computational power to train its deep neural networks, which consist of numerous interconnected layers of neurons [44].

In contrast, traditional machine learning encompasses a broader range of techniques, including algorithms like decision trees, support vector machines, and random forests. These methods typically require domain expertise to manually craft features before the learning process begins. While ML performs well with smaller datasets and simpler problems, it struggles with complex, high-dimensional data, where feature engineering becomes more challenging [45]. DL, on the other hand, excels in such scenarios due to its ability to automatically extract relevant patterns. Furthermore, ML models are often more interpretable, as algorithms like decision trees or linear regression provide insight into how features influence the final outcome. Deep learning models, by comparison, are often referred to as “black boxes” due to their complexity and the difficulty in interpreting the learned representations. Therefore, while both ML and DL aim to learn from data, DL’s architecture and ability to handle complex, unstructured data set it apart, especially for tasks in areas such as image recognition, speech processing, and natural language understanding [46].

Deep learning is revolutionizing kidney transplantation by enhancing precision at multiple stages, from donor selection to postoperative management. One critical application lies in the field of renal pathology [47,48].

Traditional histopathological evaluations of donor kidneys are subject to variability and may lead to inappropriate discards of potentially viable organs. Recent studies have developed deep learning-based models to improve the accuracy and consistency of histological assessments, particularly in frozen sections of hematoxylin and eosin-stained procurement biopsies. In one study, deep learning algorithms were applied to analyze whole-slide images from 2431 kidneys, allowing for the automated recognition of key renal compartments, such as glomeruli, arteries, and tubules, with a high degree of accuracy (90–96%). The model extracted abnormality features like glomerulosclerosis, arterial intimal fibrosis, and interstitial abnormalities, correlating them with pathologists’ scores and post-transplant outcomes, including graft loss and renal function. This led to the development of a Kidney Donor Quality Score (KDQS), which improved graft survival prediction and could potentially reduce unnecessary organ discard [47].

In another proof-of-concept study, convolutional neural networks (CNNs) were trained to classify allograft biopsies as normal, rejection, or other diseases using 5844 digital images. The models demonstrated reliable performance in cross-validation and external validation, showing potential as a triage tool for transplant biopsies. Deep learning models not only matched the performance of traditional assessments but also identified biopsy regions relevant to rejection, such as the tubulointerstitial area. These advances in deep learning hold promise for standardizing kidney biopsy evaluations, improving transplant outcomes, and optimizing organ utilization [48].

## 5. AI Model Summary—Variable Selection

In machine learning/deep learning, the selection of appropriate variables significantly influences the success or failure of a model, as irrelevant or poorly chosen features can lead to inaccurate predictions. This is why RQ4, which focuses on identifying key variables in predictive tasks related to kidney transplantation, is critical for ensuring robust and reliable outcomes in the research. To date, various ML models were developed on the basis of multiple variables. The examples presented below (Table 1) illustrate a notable lack of consensus on the specific variables that should be used to evaluate the risk of kidney transplant rejection. This inconsistency reflects the complexity of transplant outcomes and the various approaches employed to model these outcomes. Although there is no universally accepted set of predictors, a detailed comparison of the available algorithms (Table 2) reveals several key similarities and shared characteristics [23,27,28,36,49]. For instance, certain variables, such as donor hypertension, are consistently included in most of the algorithms, indicating their recognized importance in predicting transplant outcomes.

Furthermore, many of the variables frequently utilized in these models correspond to clinical parameters that are routinely measured in both kidney transplant recipients and donors as part of the standard transplantation protocol. These include factors such as the donor and recipient age, kidney function markers (e.g., serum creatinine levels), and the presence of comorbid conditions like diabetes or cardiovascular disease. The fact that these variables are regularly collected during every transplant procedure highlights their accessibility and practicality for use in predictive modeling [23,27,28,36,49]. This accessibility is critical, as it enables the seamless integration of these variables into machine learning algorithms without the need for additional or specialized data collection.

The variation in the selection of important features across different models likely stems from several factors. One significant reason is the broad range of machine learning techniques available for feature selection and model development. For example, as previously mentioned, there are numerous algorithms (e.g., Boruta and RSF) —each with distinct methods for identifying relevant variables—leading to variation in the predictors chosen. Additionally, the heterogeneity of the populations studied may further contribute to this variability [19,21]. Differences in demographic, genetic, and clinical characteristics across different transplant cohorts can influence which features emerge as the most predictive within a given population.

In some of the presented studies, such as those conducted by Mark et al. and Naqvi et al., advanced analytical methods like the Breiman–Cutler permutation importance measure were employed to evaluate the significance of various clinical variables in predicting outcomes [28,36]. The Breiman–Cutler method, widely utilized in machine learning, is particularly associated with random forests. This method provides insights into the relative importance of each variable by assessing the change in model accuracy when the values of a given variable are randomly permuted or shuffled. Specifically, the method works by systematically altering the values of one feature while leaving others unchanged. If the randomization of a feature results in a significant drop in the model’s predictive accuracy, that feature is considered important because its correct values were crucial to making accurate predictions. On the other hand, if permuting a variable leads to little or no change in performance, that variable likely has less predictive power [36]. Using this methodology, in was possible to identify key factors that significantly influence long-term graft survival. Among the most important variables were the presence of diabetes in the recipient and the primary cause of renal failure leading to the need for transplantation. The presence of diabetes, for example, was found to correlate strongly with adverse transplant outcomes, likely due to the broader systemic effects of the disease, which can affect immune function and increase the risk of complications. Similarly, the underlying cause of kidney failure, whether it was due to conditions such as glomerulonephritis, hypertension, or polycystic kidney disease, played a critical role in determining the long-term success of the transplant [28,36,50].

## 6. Discussion—Challenges, Limitations, and Future Directions

As shown in Table 1, and as determined by the Breiman–Cutler permutation importance assessment, the most critical factors associated with the risk of transplant rejection were recipient comorbidities, such as diabetes and the primary cause of kidney failure. Additionally, other frequently observed factors in the included studies were the recipient and donor age, donor hypertension, donor cause of death, and the duration of dialysis before transplantation [28,36].

In conventional studies conducted without the use of AI which aimed to identify risk factors for kidney transplant rejection, several significant variables were often reported. These include, most of all, the recipient and donor age, HLA mismatches, PRA value, recipient’s smoking addiction, number of prior kidney transplantations, history of thromboembolism, prolonged dialysis preceding the transplantation, recipient’s various comorbidities, e.g., diabetes mellitus and hypertension, BK viremia, etc. [51,52,53,54].

The findings obtained through traditional methodologies exhibit notable similarities to those derived using AI, providing additional validation for this advanced technology. However, the selection of variables in studies is often subjective and inconsistent across different investigations. This inconsistency creates gaps that hinder the identification of patterns and correlations between patients’ populations. We hypothesize that AI may be found useful in that field by integrating clinical data from various clinical centers, making data more widely available, more detailed, and, as a consequence, more reliable.

Moreover, we demonstrated that AI may find applications not only in predicting kidney graft survival but also in other various aspects of transplantology, such as donor–recipient pairing, immunosuppressive dosage estimation, or virtual biopsy. However, on the other hand, there are numerous challenges and limitations of AI use which have to be underlined as well, which we will discuss further.

### 6.1. Challenges and Limitations

Machine learning models, while demonstrating significant potential in the field of transplantology, encounter distinct limitations that can critically affect their effectiveness, particularly in clinical applications. One of the most prominent issues is overfitting, a phenomenon where the model not only captures the intrinsic patterns in the training data but also memorizes noise and outliers. This results in models that perform well on training datasets but exhibit poor generalization to unseen data, ultimately compromising the reliability of predictions in real-world clinical scenarios. Overfitting is especially prevalent in complex models with a high number of parameters, where the risk of encoding irrelevant information is substantially increased. Another major limitation stems from the reliance on retrospective data, which is often sourced from single institutions or homogenous patient populations. This can introduce selection bias, as the data may not adequately represent the heterogeneity of the broader population, thereby limiting the model’s generalizability. For example, a model developed using data from a specialized medical center may perform suboptimally in community hospital settings, where patient demographics and clinical practices diverge. Moreover, many studies do not perform robust external validation across diverse settings, a critical step for evaluating the model’s performance in varied clinical environments. Without such validation, the clinical utility of these models remains uncertain and potentially misleading [55,56].

Additionally, model validation practices in ML studies are frequently insufficient. Simplistic validation techniques, such as basic train–test splits, are often employed, which can result in overly optimistic performance metrics. More rigorous validation methods, like k-fold cross-validation or external validation on independent datasets, are less frequently utilized, reducing the confidence in the reported accuracy and reliability. Compounding this issue is the inadequate reporting of validation protocols in many ML studies, hindering the reproducibility and the potential for further advancement of the field by other researchers. A key challenge in ML is model interpretability, particularly with complex architectures such as deep learning networks. While these models can achieve a high predictive accuracy, their “black box” nature obscures the underlying decision-making processes, making it difficult for clinicians to understand or trust the model’s outputs [38,39]. This lack of transparency can impede clinical acceptance, as healthcare professionals require clear, interpretable rationales for predictions in order to make informed decisions. Furthermore, the inability to interpret model decisions complicates the identification of biases or errors in the predictions, which can undermine the model’s clinical credibility and reliability. Ethical concerns, particularly related to bias in training data, further exacerbate these limitations. If the datasets used to train ML models are not representative of diverse populations, the resulting models may perpetuate or even exacerbate existing healthcare disparities. For instance, models trained predominantly on data from one demographic group may yield suboptimal performance for under-represented groups, leading to inequitable healthcare outcomes. Addressing these limitations—such as improving data representativeness, implementing robust validation strategies, enhancing interpretability, and minimizing bias—remains essential for the successful integration of ML into clinical practice, necessitating continuous research and development to strengthen model robustness and clinical applicability [55,56].

### 6.2. Possible Future Directions

The future application of AI in kidney transplantology may significantly advance the field of xenotransplantation, making it a more accessible and efficient therapeutic option and, in consequence, increase organ availability for patients requiring renal replacement therapy. Solez and Eknoyan have already emphasized the potential role of AI in xenotransplant pathology, particularly in the analysis of complex histopathological data and the optimization of diagnostic accuracy [57]. Notably, AI-driven approaches are currently under scrutiny in heart xenotransplantation, where they facilitate donor–recipient matching, assess immune compatibility, and enable predictive modeling to reduce the risk of graft rejection, which can be possibly utilized in the kidney xenotransplantation in the future [58].

## 7. Conclusions

This review has provided comprehensive answers to the research questions posed. The findings revealed the various machine learning algorithms and techniques (RQ1) applied in kidney transplantation, along with specific application areas (RQ2) where these methods have been utilized. Additionally, the review identified the most appropriate method for achieving accurate results in the intended analysis (RQ3). Finally, the key variables commonly used in predictive models related to kidney transplantation (RQ4) have been thoroughly outlined. These answers are presented and discussed in detail in the previous sections of this paper.

In conclusion, the integration of AI technologies in kidney transplantation presents a promising avenue for enhancing patient outcomes through improved predictive modeling and personalized treatment strategies. As we demonstrated, AI may be an effective tool in predicting the graft survival, immunosuppressive agent dosage estimation, virtual biopsy, or donor–recipient pairing. To date, a great number of factors associated with an increased risk of kidney transplant rejection have been identified using traditional statistical methods, as well as through the application of artificial intelligence. Yet, we hypothesize that numerous unknown variables and their hidden interactions, which may be exceptionally challenging to detect using traditional methods, can significantly influence the predictions and treatment outcomes in patients following kidney transplantation. In the future, AI has the potential to empower researchers to identify and comprehensively investigate these factors and their interactions. However, addressing challenges such as data quality, algorithmic bias, and the need for model interpretability is crucial for the successful implementation of these advanced tools in clinical practice. Continued research and collaboration among clinicians and associate professionals will be essential to fully realize the benefits of AI in this field.

## Figures and Tables

**Table 1:** An overview of available ML models to predict kidney rejection.

AIM, STUDY DESIGN	VARIABLES	OUTCOMES	AUTHORS, YEAR, COUNTRY
Prediction of kidney graft survival—Original retrospective research	Model for ages 50 and under: CDD, CIT, CREA, DDM, DHT, ETH, HCV, KFR, PS, RA, REG, RDM, RFS, RMC.	The proposed models outperformed the US kidney allocation system’s Estimated Post-Transplant Survival (EPTS) model and other recent models, achieving a five-year concordance index of 0.724 compared to 0.697 for the EPTS. Key variables (per the Breiman–Cutler permutation importance) included the RA, RDM and KFR	Mark et al., 2019, USA, [28]
	Model for ages 51 and older: CDD, CIT, COPD, CREA, DA, DHT, DIA, ETH, HCV, KFR, RA, RDM, RFS.		
Facilitation and improved prediction of kidney transplant survival for individual donor–recipient pairs—Original retrospective research	CDD, COPD, DA, DBMI, DBT, DCREA, DDM, DETH, DHT, DHCV, DIA, DG, DSM, ETH, HLA, KFR, RA, RAI, RBMI, RBT, RDM, RG, RFS, RMC, RWT	The model enabled the accurate prediction of kidney transplant survival, offering critical support to medical professionals and transplant candidates in identifying the optimal donor–recipient pairing	Paquette et al., 2022, USA, [27]
To estimate the risk of kidney graft failure across three distinct temporal cohorts (within 1 year, within 5 years, and beyond 5 years post-transplant), based on the clinical and demographic characteristics of both donor and recipient—Original retrospective research	CDD, CIT, DA, DBMI, DCREA, DDM, DETH, DG, DHCV, DHT, DIA, DRC, ETH, HLA, KFR, PKT, PRA, RA, RBMI, RDM, RFS, RG, RHT, RMA, RVD, TT	ML algorithms can effectively predict graft survival by leveraging donor and recipient factors that are routinely collected during standard clinical care. Most important features included: HLA, DIA, KFR, and RDM	Naqvi et al., 2021, USA, [36]
To develop a risk index for application in pre-transplant decision-making processes—Original retrospective research	Cox model: DA, DHT, DIA, KFR, RA, RVD.	The methods demonstrate a moderate ability to differentiate patients at higher risk of graft failure and provide predictions of graft failure with a moderate level of accuracy	Senanayake et al., 2021, Australia, [49]
	RSF model: CDD, DA, DCREA, DBMI, DDM, DETH, DIA, DHT, HLA, KFR, OG, PKT, PTX, RA, RSM, RVD		
To develop a predictive model capable of accurately identifying kidneys at risk of being discarded—Original retrospective research	CDD, DA, DBMI, DBT, DCREA, DDM, DETH, DGC, DHT, DHCV, DMIN, DMA, DSM, DTTA, HBCA, HBSA, HBSAB, HTLV	Machine learning techniques, such as random forest, can enhance the accuracy of identifying kidneys at risk of discard	Barah et al., 2021, USA, [23]

(CDD (cause of donor death), CIT (cold ischemia time), COPD (recipient chronic obstructive pulmonary disease), CREA (recipient creatinine at the time of transplantation), DA (donor age), DBMI (donor body mass index), DBT (donor blood type), DCREA (donor creatinine level), DDM (donor diabetes), DETH (donor ethnicity), DG (donor gender), DHCV (donor HCV status), DHT (donor hypertension), DIA (recipient dialysis), DMA (donor malignancy), DMIN (donor history of myocardial infarction), DRC (donor and/or recipient CMV status), DSM (donor nicotine addiction), DTTA (donor tattoos), ETH (recipient ethnicity), HBCA (donor hepatitis b core antibody), HBSA (donor hepatitis b surface antigen), HBSAB (donor hepatitis b surface antibody), HCV (recipient HCV status), HLA (number of HLA A, B, or DR mismatches), HTLV (donor human t lymphocyte virus), KDPI (kidney donor predictive index), KFR (kidney failure primary reason), OG (oliguria), PKT (previous kidney transplantation), PRA (peak panel reactive antibody), PS (payment source), PTX (pre-emptive transplant), RA (recipient age), RAI (recipient angina indicator), RBMI (recipient body mass index), RBT (recipient blood type), REG (region of transplantation), RG (recipient gender), RFS (recipient functional status at the moment of transplantation), RHT (recipient hypertension), RMA (recipient malignancy), RMC (recipient medical condition at transplant (hospitalized or not)), RSM (recipient nicotine addiction), RVD (recipient vascular disease), RWT (recipient waiting time for transplantation), TT (transplant type)).

**Table 2:** ML models variables overview.

Variable	Mark et al. 2019 (Ages 50 and Under) [28]	Mark et al. 2019 (Ages 51 and Above) [28]	Paquette et al. 2022 [27]	Naqvi et al. 2021 [36]	Senanayake et al. (Cox) 2021 [49]	Senanayake et al. (RSF) 2021 [49]	Barah et al. 2021 [23]
CDD	+	+	+	+		+	+
COPD		+	+				
CREA	+	+					
CIT	+	+		+			
DA		+	+	+	+	+	+
DBMI			+	+ (weight and height analyzed separately)		+ (only height)	+ (weight and height analyzed separately)
DBT			+				+
DDM	+		+	+		+	+ (type)
DETH			+	+		+	+
DCREA			+	+		+	+
DHCV			+	+			+
DG			+	+			+
DHT	+	+	+	+	+	+	+
DIA		+	+	+	+	+	
DMA							+
DMIN							+
DRC				+			+ (only donor)
DSM			+				+
DTTA							+
ETH	+	+	+	+			
HBCA							+
HBSA							+
HBSAB							+
HCV	+	+					
HLA			+	+		+	
HTLV							+
KDPI							+
KFR	+	+	+	+	+	+	
OG						+	
PKT				+		+	
PRA				+			
PS	+						
PTX						+	
RA	+	+	+	+	+	+	
RAI			+				
RBMI			+	+ (weight and height analyzed separately)			
RBT			+				
REG	+						
RG			+	+			
RFS	+	+	+	+			
RHT				+			
RMA				+			
RMC	+		+				
RSM							+
RVD				+	+	+	
RDM	+	+	+ (type)	+			
RWT			+				
TT				+			

(CDD (cause of donor death), CIT (cold ischemia time), COPD (recipient chronic obstructive pulmonary disease), CREA (recipient creatinine at the time of transplantation), DA (donor age), DBMI (donor body mass index), DBT (donor blood type), DCREA (donor creatinine level), DDM (donor diabetes), DETH (donor ethnicity), DG (donor gender), DHCV (donor HCV status), DHT (donor hypertension), DIA (recipient dialysis), DMA (donor malignancy), DMIN (donor history of myocardial infarction), DRC (donor and/or recipient CMV status), DSM (donor nicotine addiction), DTTA (donor tattoos), ETH (recipient ethnicity), HBCA (donor hepatitis b core antibody), HBSA (donor hepatitis b surface antigen), HBSAB (donor hepatitis b surface antibody), HCV (recipient HCV status), HLA (number of HLA A, B, or DR mismatches), HTLV (donor human t lymphocyte virus), KDPI (kidney donor predictive index), KFR (kidney failure primary reason), OG (oliguria), PKT (previous kidney transplantation), PRA (peak panel reactive antibody), PS (payment source), PTX (pre-emptive transplant), RA (recipient age), RAI (recipient angina indicator), RBMI (recipient body mass index), RBT (recipient blood type), REG (region of transplantation), RG (recipient gender), RFS (recipient functional status at the moment of transplantation), RHT (recipient hypertension), RMA (recipient malignancy), RMC (recipient medical condition at transplant (hospitalized or not)), RSM (recipient nicotine addiction), RVD (recipient vascular disease), RWT (recipient waiting time for transplantation), TT (transplant type)).

## Data Availability

Not applicable.
